# The Role of Just World Beliefs in Responding to the COVID-19 Pandemic

**DOI:** 10.1007/s11211-022-00388-1

**Published:** 2022-02-17

**Authors:** Antonia Mariss, Nina Reinhardt, Simon Schindler

**Affiliations:** Department of Psychology, University of Kassel, Holländische Straße 36-38, 34127 Kassel, Germany

**Keywords:** Belief in a just world, Social distancing, COVID-19, Risk perception, Empathy

## Abstract

This study investigated whether people’s personal belief in a just world (BJW) is linked to their willingness to physically distance themselves from others during the COVID-19 pandemic. Past research found personal BJW to be positively related to prosocial behavior, justice striving, and lower risk perceptions. If social distancing reflects a concern for others, high personal BJW should predict increased interest in social distancing. If social distancing reflects a concern for one’s personal risk, high personal BJW should predict decreased interest in social distancing. Results of a pre-registered internet-based study from Germany (*N* = 361) indicated that the higher people’s personal BJW, the more they generally practiced social distancing. This association still occurred when controlling for empathy, another significant predictor of social distancing. There were no mediation effects of empathy and risk perception. The findings extend knowledge on the correlates of social distancing in the COVID-19 pandemic which could be used to increase compliance among citizens.

## Introduction

To slow the spread of the coronavirus disease COVID-19, people’s compliance with implemented prevention measures is very important (e.g., Sailer et al., 2020). One of the most common measures is *social distancing*; meaning that people physically minimize interpersonal contacts for instance by avoiding crowds or interactions with others apart from one’s own household (Centers for Disease Control and Prevention, 2021). This reduces the risk that an infected individual will transmit the virus directly to others. Social distancing thus aims at protecting oneself from a potential infection, but also at protecting the health of others (Favero & Pedersen, 2020).

Research has investigated possible predictors and correlates of social distancing, such as, e.g., the Big 5 personality traits (Blagov, 2021) and political orientation (Kushner Gadarian et al., 2020). Among others, social distancing has also been linked to more trust in governments (Han et al., 2021), higher rule compliance (Twardawski et al., 2021), and higher moral support of the measures and a greater perception of descriptive social norms (van Rooij et al., 2020). Furthermore, research indicated that people’s social distancing behavior can be predicted by their empathy toward those most vulnerable to COVID-19 (Pfattheicher et al., 2020) and that people show higher compliance with COVID-19 measures like social distancing, the higher they perceive the risk of COVID-19 (e.g., Barrios & Hochberg, 2020; Oosterhoff & Palmer, 2020; Plohl & Musil, 2020; Wise et al., 2020). To increase people’s willingness to socially distance themselves from others, research found messages targeting prosocial motives (e.g., Blagov, 2021; Heffner et al., 2020) as well as messages targeting the threat of COVID-19 (Heffner et al., 2020) to be effective. Another possible predictor of social distancing could be people’s belief in a just world (BJW).

### Belief in a Just World

The concept of just world beliefs claims that people have the need to believe that the world is just in a way that people get what they deserve and that they equally deserve what they get (Lerner, 1977, 1980; Lerner & Miller, 1978). That way, people create a stable, orderly, and predictable environment for themselves where they feel safe and confident about their future (Lerner, 1980; Lerner & Miller, 1978). To be part of this, people with a high belief in a just world feel obligated to behave fairly and to restore justice themselves, indicating a social contract between the world and themselves (Dalbert, 2002; Sutton et al., 2017).

A distinction between two facets of BJW has been made by Dalbert (1999, 2009) who differentiated between a *personal BJW*—that is, believing that the world is just for oneself—and a *general BJW*—that is, believing that the world is a just place in general. Similarly, Lipkus et al. (1996) differentiated between a belief in just world for the self (BJW-S) and a belief in a just world for others (BJW-O). Altogether, the more general facets of BJW—notably BJW-O—have rather been linked to negative other-oriented variables such as more social discrimination and harsher social attitudes toward those suffering from injustice (e.g., Bègue & Bastounis, 2003; Hafer & Sutton, 2016; Sutton & Douglas, 2005). In contrast, personal BJW and BJW-S have rather been associated with more desirable intentions and behaviors such as prosocial behavior (Bègue, 2014; Bègue & Bastounis, 2003; Bègue et al., 2008; Bègue et al., 2008; Bierhoff et al., 1991; Dalbert, 1999, 2001; Hafer & Sutton, 2016; Schindler et al., 2019), but also with other positive self-oriented variables including a higher subjective well-being, mental health, life satisfaction, emotional stability, and low levels of stress (e.g., Bartholomaeus & Strelan, 2019; Bègue & Bastounis, 2003; Hafer & Sutton, 2016; Lipkus et al., 1996; Sutton & Douglas, 2005; Sutton & Winnard, 2007; Sutton et al., 2008).

Additionally, just world beliefs have been linked to altered risk perceptions: People with a high BJW feel less vulnerable to negative events given they perceive themselves as good (Furnham, 2003; Hafer et al., 2001; Lambert et al., 1999). Hence, they engage more in high-risk intentions and behaviors (e.g., Becker, 1974; Hafer et al., 2001). Such a mediating effect of personal risk perception on the relationship between BJW and high-risk behavior has already been shown in the health-related domain (Riley & Baah-Odoom, 2012) but it remains questionable if these findings can be transferred to the COVID-19 pandemic.

### The Present Research

People’s social distancing behavior during the pandemic can be viewed from two perspectives: On one hand, given the protection for other people’s health, social distancing reflects a concern for others and hence a prosocial behavior to protect other people’s health (e.g., Favero & Pedersen, 2020; Twardawksi et al., 2021). On the other hand, given the self-protective component, social distancing reflects a concern for oneself to reduce one’s own risk. Both aspects─prosocial behavior and self-protection─primarily address personal BJW. In our study, it is therefore evident to specifically focus on the role of personal BJW in social distancing (in contrast to general BJW or a mixed measure like, e.g., Devereux et al., 2021).

In line with the two perspectives on social distancing, two distinct scenarios are conceivable and reflected in our two opposing main hypotheses. First, assuming social distancing during the COVID-19 pandemic to be a prosocial behavior and a matter of justice is in line with recent research on people’s compliance with COVID-19 prevention measures (e.g., Jordan et al., 2020; Pfattheicher et al., 2020; Twardawski et al., 2021). According to the justice motive, several forms of prosocial behavior have already been positively linked to believing in a just world for oneself (e.g., Bègue, 2014; Bègue & Bastounis, 2003; Bègue et al., 2008); thus, to people’s personal BJW. Consequently, we hypothesize people’s personal BJW to be positively linked to self-reported social distancing (*Hypothesis 1*). Furthermore, recent research already showed empathy for vulnerable others to be positively linked to social distancing during the COVID-19 pandemic (Pfattheicher et al., 2020). Even not preregistered, it is plausible to assume that people’s personal BJW predicts social distancing independently of empathy (*Hypothesis 2*).

From the second perspective, social distancing can primarily be regarded as a measure of self-protection against a COVID-19 infection and therefore dependent on people’s perception of their own risk of infection. Because past research indicates people high in their BJW to feel less vulnerable to negative events (e.g., Furnham, 2003; Lambert et al., 1999), they might also perceive their personal risk of a COVID-19 infection as low and engage in less social distancing (e.g., Barrios & Hochberg, 2020; Plohl & Musil, 2020; Wise et al., 2020). Contrary to the first hypothesis, we alternatively predict people’s personal BJW to be negatively linked to social distancing in times of the COVID-19 pandemic (*Hypothesis 3*).

Additionally, as a more explorative approach, we aim to address potential factors responsible for the underlying processes of our two main hypotheses (Hypotheses 1 and 3). Given the assumption that people’s BJW affects their risk perception and since risk perception has been shown to be linked to less compliance with prevention measures (e.g., Oosterhoff & Palmer, 2020; Plohl & Musil, 2020; Wise et al., 2020), risk perception seems to be a potential mediator of the association between personal BJW and social distancing. Another conceivable possibility is that empathy (partially) mediates the linkage between personal BJW and social distancing (see derivation of Hypotheses 1 and 2). In sum, we aim to test a mediation via multiple mediators (i.e., risk perception and empathy) for the association between personal BJW and social distancing (*Hypothesis 4*); please note that this is different to our preregistration protocol, wherein we suggested to only test risk perception as single mediator.

As explained above, all our hypotheses focus on people’s personal BJW. However, in line with Bartholomaeus and Strelan (2019), who recommend testing both BJW facets for their independent effects, we added further analyses for all hypotheses with people’s general BJW as predictor when controlling for personal BJW. As we did not expect any significant associations, these statistical analyses served as an exploratory refinement of our preregistered hypotheses. Hence, all additional results, data, the material of the study, and a more detailed sample description are available in the Supplemental Material (see https://osf.io/yz2cg/?view_only=921b5eb4fcb4482c898e7457382f99a1).

## Method^1^

### Participants

Data were collected online between April 22nd and May 3rd, 2020, while social distancing had been implemented as a rule throughout Germany (Presse und Informationsamt der Bundesregierung, 2020). With an assumed power of 90%, setting Type I error rate at *p* < 0.05, and assuming a small to medium effect size of *r* = 0.25, an a priori power analysis for correlation (two-tailed) revealed a minimum sample size of *N* = 160. Our resources enabled us to collect data from 367 participants—recruited via personal networks and social media—which we have exhausted to detect even smaller effects. Despite oversampling, we did not analyze any data prior to stopping data collection.

After excluding *n* = 6 participants who did not agree in the declaration of consent, the final sample consisted of *N* = 361 participants (*M*_age_ = 33.37, *SD*_age_ = 16.05, range: 14–87; 67% females, 32% males, 0.28% diverse; 97% living in Germany; 48% university students). With our final sample size, we were able to find a significant small effect of *r* = 0.17 with 90% power. Regarding their *personal experiences with COVID-19*, five participants (1%) reported to have been infected themselves, 62 participants (17%) stated to know someone who has been infected and three participants (1%) stated to know someone who had died from COVID-19. Additionally, 69 participants (19%) considered themselves at higher risk of infection.

### Procedure and Measures

First, we assessed participants’ personal BJW with seven items and general BJW as a control variable with six items created by Dalbert (1999). Participants responded on a 6-point scale ranging from 1 (*strongly disagree*) to 6 (*strongly agree*).

Next, we measured personal risk perception of COVID-19 with two novel items concerning participants’ *perceived infection probability* and their *perceived infection severity* (see the Supplemental Material). Participants responded on a 7-point scale ranging from 1 (*very unlikely/not severe at all*) to 7 (*very likely/very severe*). Given the low internal consistency of the combined measure (*risk perception*; *α* = 0.12)—unlike preregistered—we focused the statistical analyses on *perceived infection probability* because it is the item most directly related to social distancing. Results of the combined measure are reported in the Supplemental Material (see Further Results Section 3). Additionally, we included one item concerning *threat perception* to check whether our sample agreed that COVID-19 is a threat with the potential to harm lives, measured on a 7-point scale ranging from 1 (*totally disagree*) to 7 (*totally agree*).

Subsequently, participants’ social distancing behavior during the pandemic was assessed with two 5-point scales created by Pfattheicher et al. (2020) ranging from 1 (*strongly disagree/very unlikely*) to 6 (*strongly agree/very likely*). The first scale which we refer to as *general social distancing* included two items on participants’ own behavior (“Because of coronavirus COVID-19, I am massively curtailing social contact [so-called social distancing].”) and on their attitude toward other people’s behavior (“Because of coronavirus COVID-19, it is very important that others massively curtail social contact [so-called social distancing].”). The second scale, referred to as *concrete social distancing*, included five items concerning participants’ own planned behavior (e.g., “During the next days, I will meet friends outside of my apartment.”). Upon retrospective reflection and only given the moderate internal consistency of the second scale (*α* = 0.63), we do not consider this scale as accurate enough for reliably measuring people’s social distancing behavior (for a more detailed discussion see Limitations). Consequently, results of this measure will only be reported shortly within the correlation matrix (for all additional analyses with this concrete social distancing measure see the Supplemental Material, Further Results Section 3).

Participants’ *empathy* toward vulnerable others was measured with three items created by Pfattheicher et al. (2020; e.g., “I am very concerned about those most vulnerable to coronavirus COVID-19.”) on a 5-point scale ranging from 1 (*strongly disagree*) to 5 (*strongly agree*). In accordance with Pfattheicher et al., three filler items were added to reduce demand characteristics.

Last, participants indicated their personal experiences with COVID-19 and provided demographic data (age, gender, highest educational attainment, current occupational status, and place of living).

## Results

Statistical analyses were conducted in line with our preregistered analysis plan. To get a more extensive understanding of the data, we additionally conducted some explorative analyses which were not preregistered but which will be clearly marked as such. Since Shapiro–Wilk tests revealed that none of the variables followed a normal distribution (all *p*s < 0.001), correlations were calculated using Spearman’s rho correlation coefficients (Spearman, 1904). Table 1 shows means, standard deviations, and Spearman correlations among all variables with Cronbach’s alpha in the diagonal. For all following analyses including the variable *gender* (0 = female, 1 = male), we excluded *n* = 1 participant who identified as gender diverse.

**Table 1:** Means, standard deviations, and Spearman correlations among all variables assessed in this study with Cronbach’s alpha in the diagonal

Variable	1	2	3	4	5	6	7	8	9	10	*M*	*SD*
1. Personal BJW	.89										4.36	0.92
2. General BJW	.29***	.80									2.42	0.82
3. General social distancing^a^	.14*	.04	.78								4.41	0.72
4. Concrete social distancing^a^	.05	.05	.40***	.63							3.92	0.84
5. Threat perception	.13*	− .09	.36***	.13*	–						6.13	1.27
6. Perceived probability	− .06	− .05	.07	− .01	.10	–					3.96	1.50
7. Perceived severity	− .08	.04	.16**	.20***	.13*	.07	–				2.97	1.45
8. Empathy	.08	− .05	.28***	.08	.18***	.15**	.12*	.87			4.32	0.75
9. Gender^b^	.00	.08	− .03	− .02	.04	− .05	− .00	− .13*	–	–	–	–
10. Age	− .08	.06	.04	.07	.02	− .07	.45***	.04	.17*	–	33.38	16.07

*N* = 360. BJW = belief in a just world

^a^Social distancing with higher values indicating more social distancing.

^b^0 = female and 1 = male

*p < .05. **p < .01. ***p < .001

The results of the correlation matrix show first evidence for Hypothesis 1 by revealing a significant positive correlation between personal BJW and general social distancing (*r*_s_ = 0.14, *p* = 0.011), indicating that participants high in their personal BJW show more social distancing. Taken the items of the general social distancing measure separately, only the item assessing participants’ attitude on other people’s behavior was significantly linked to personal BJW (*r*_s_ = 0.15, *p* = 0.004) and not the item concerning their own general behavior (*r*_s_ = 0.10, *p* = 0.066). None of the two items of general social distancing was linked to general BJW (both *p*s > 0.356). No significant correlation between personal BJW and concrete social distancing was found (*r*_s_ = 0.05, *p* = 0.380); actually, none of the items from this scale was significantly correlated with personal BJW (all *p*s > 0.247; see Further Results Section 3.2 in the Supplemental Material).

### Personal BJW and Social Distancing

For a more detailed test of our hypotheses, we conducted hierarchical multiple regression analyses on general social distancing (for regression analyses on concrete social distancing see the Supplemental Material, Further Results Section 3.3). Because our hypotheses were solely built on people’s personal BJW, we used personal BJW as a first predictor (Model 1) and gradually added general BJW as control variable (Model 2). To explore whether the effect of personal BJW remained robust (Hypothesis 2), we additionally added empathy as control variable (Model 3). Table 2 shows that Model 3 explained 12.10% (11.40% adjusted) of the variance, *F*(3, 356) = 16.33, *p* < 0.001, and that empathy was a significant positive predictor, *B* = 0.23, *t*(356) = 6.45, *p* < 0.001. Most importantly, personal BJW was also a significant positive predictor in Model 3, *B* = 0.08, *t*(356) = 2.02, *p* = 0.044. This indicates that participants high in their personal BJW show more social distancing, even when controlling for empathy and general BJW; thus, supporting Hypotheses 1 and 2.

**Table 2:** Hierarchical multiple regression analysis on general social distancing

Variable	*B*	*SE*	β	*R*^*2*^	*∆R*^*2*^
*Model 1*				.02 (.02)	
Personal BJW	0.11	0.04	0.15***		
*Model 2*				.02 (.02)	< .001
Personal BJW	0.10	0.04	0.14*		
General BJW	0.01	0.04	0.01		
*Model 3*				.12 (.11)	.10***
Personal BJW	0.08	0.04	0.11*		
General BJW	0.03	0.04	0.04		
Empathy	0.23	0.04	0.32***		
*Model 4*				.13 (.11)	.004
Personal BJW	0.08	0.04	0.11*		
General BJW	0.03	0.05	0.05		
Empathy	0.22	0.04	0.31***		
Personal experiences^a^	0.06	0.08	0.04		
Perceived probability	0.03	0.04	0.04		
Age	< 0.001	0.002	0.003		
Gender^b^	0.03	0.08	0.02		

*N* = 360. BJW = belief in a just world. The predictors were z-standardized. Adjusted *R*^*2*^ is displayed in parentheses

^a^Personal experiences = Dummy coded variable for personal experiences with COVID-19 (0 = without personal experiences 1 = with personal experiences; see Method for operationalization)

^b^0 = female and 1 = male

^*^p < .05. **p < .01. ***p < .001

As a more explorative approach and unlike preregistered, we further added perceived infection probability, personal experiences with COVID-19 as a dummy coded predictor (0 = without personal experiences, 1 = with personal experiences), age, and gender as control variables (Model 4; see Table 2). Results show that none of these additional control variables were significant predictors (all *p*s ≥ 0.382). Again, and most importantly, personal BJW remained robust in Model 4, *B* = 0.08, *t*(352) = 2.07, *p* = 0.039, even when controlling for empathy, perceived infection probability, personal experiences, age, and gender.

### The Mediating Role of Risk Perception and Empathy

As a more explorative approach, we aim to test a mediation via multiple mediators (i.e., risk perception and empathy) for the association between personal BJW and social distancing (*Hypothesis 4*). Please note that this procedure is different to our preregistration protocol, wherein we only planned to test risk perception as a single mediator. We applied Model 4 of the Process macro of Hayes (2013), using personal BJW as the independent variable and (a) perceived infection probability and (b) empathy as two parallel mediators. Applying nonparametric bootstrapping, we conducted the analysis with general social distancing as the dependent variable and found no mediation effects, as the confidence intervals for the indirect effects included zero (see Table 3), thus opposing Hypothesis 4. See the Supplemental Material (Further Results Section 3.4) for the same mediation analyses but using concrete social distancing as dependent variable, general BJW as predictor, and the combined measure of risk perception as mediator, respectively.

**Table 3:** Regression coefficients of the mediation analysis with two parallel mediators on the relationship between personal belief in a just world (BJW) and general social distancing

Independent variable (*X*)	Mediator variable (*M*)	Dependent variable (*Y*)	*a*	*b*	c′	c	*ab*
Personal BJW		General social distancing			0.09*	0.09*	0.02 (− 0.01; 0.06)
	Perceived probability		− 0.02	0.03			− 0.001 (− 0.08; 0.004)
	Empathy		0.08	0.22***			0.02 (− 0.01; 0.06)

*N* = 361. The predictors were z-standardized. *a* = effect of *X* on *M*; *b* = effect of *M* on *Y*; *c*’ = direct effect of *X* on *Y* controlling *M*; *c* = total effect on *Y*; *ab* = indirect effect of *X* on *Y* through *M (mediation).* Regression coefficients are unstandardized (*B*). 95% bias-corrected confidence intervals are shown in square bracket

*p < .05. **p < .01. ***p < .001

### Further Analyses

In line with our preregistration, we calculated correlation coefficients among all variables again to explore whether results changed when excluding the 119 participants who reportedly have had personal experiences with COVID-19. We assumed that when participants belonged to a risk group, have been infected themselves or know someone who has been infected or died, their perception of COVID-19 and behavior might change in comparison with people who have not yet had such points of contact. The reduced sample consisted of *N* = 242 participants (*M*_age_ = 29.80, *SD*_age_ = 12.73, range: 14–80; 68% females, 31% males, 0.27% diverse). Compared to the original sample (*r*_s_ = 0.14, *p* = 0.011), participants’ personal BJW did not significantly correlate with general social distancing (*r*_s_ = 0.12, *p* = 0.075). As the correlation coefficient did not decrease much, this could be due to decreased power in the reduced subsample. There was also no relationship between personal BJW and concrete social distancing (*r*_s_ = 0.02, *p* = 0.789). General BJW significantly correlated neither with general social distancing nor with concrete social distancing (all *ps* > 0.825).

Although not preregistered, we also calculated correlations for only those who have had personal experiences with COVID-19 (*N* = 119, *M*_age_ = 40.62, *SD*_age_ = 19.38, range: 18–87; 65.5% females, 34.5% males). In line with the original sample, participants’ personal BJW significantly correlated with general social distancing (*r*_s_ = 0.17, *p* = 0.043), but not with concrete social distancing (*r*_s_ = 0.11, *p* = 0.206). Again, general BJW was neither significantly correlated with general social distancing nor with concrete social distancing (all *ps* > 0.103). See the Supplemental Material (Further Results Section 3.6) for *t* tests between the two subsamples.

As a further explorative analysis—which was not preregistered—we tested for a moderating effect of perceived infection probability on the association between personal BJW and social distancing. Using the Process macro of Hayes (2013), we found a significant interaction of personal BJW and perceived infection probability on general social distancing, *t*(357) = − 2.17, *B* = − 0.06, *SE* = 0.03, *p* = 0.031. Among people with a low perceived infection probability (− 1 SD below mean), those with a strong personal BJW showed increased general social distancing compared to people with a weak personal BJW*, t*(357) = 3.55, *B* = 0.20, *SE* = 0.06, *p* < 0.001. Among people with a high perceived probability (+ 1 SD above mean), personal BJW did not predict general social distancing, *t*(357) = 0.22, *B* = 0.01, *SE* = 0.06, *p* = 0.823. There was no significant interaction between personal BJW and perceived probability on concrete social distancing nor between personal BJW and perceived infection severity/the combined measure of risk perception, respectively, on neither general nor concrete social distancing (all *p*s > 0.154; see Further Results Section 3.5 in the Supplemental Material).

## Discussion

There were two possible but contrary scenarios claiming either a positive relationship (Hypothesis 1) or a negative relationship (Hypothesis 3) between personal BJW and social distancing. Our data provided initial support for Hypothesis 1 (and not for Hypothesis 3) insofar that the higher people’s personal BJW, the more they reported to generally engage in social distancing. This effect remained stable when controlling for general BJW and empathy toward vulnerable others; thus, supporting Hypothesis 2. The effect also remained stable when controlling for participants’ age, gender, perceived infection probability, and their personal experiences with COVID-19. Besides, there was no evidence that empathy or risk perceptions mediate the relationship between personal BJW and social distancing; thus, opposing Hypothesis 4. Exploratorily, our moderation analyses indicated that under low perceived infection probability people high in their personal BJW showed increased general social distancing compared to people low in their personal BJW; under high perceived infection probability personal BJW did not predict social distancing. Overall, general BJW did not predict any differences in people’s social distancing behavior and the reported effects did not occur for the concrete measure of social distancing (for a detailed discussion see limitations below).

Overall, the results and derivation of our two main hypotheses can be explained insofar that social distancing fulfils different functions and can be perceived differently (Favero & Pedersen, 2020). This is supported by recent research on the COVID-19 pandemic, providing evidence for both a self-protective and other-protective component. We expected a positive relationship between personal BJW and social distancing when protecting others and acting fairly is the primary concern (Hypothesis 1), and a negative relationship when the primary concern is one’s own risk (Hypothesis 3). Our finding of a positive relationship between personal BJW and increased social distancing is in line firstly with past research linking personal BJW to justice striving (e.g.,Dalbert, 2009; Lerner, 1977) and to prosocial intentions and behaviors (e.g., Bègue, 2014), and secondly with recent research linking such prosocial intentions to prevention behaviors in the COVID-19 pandemic (e.g., Capraro & Barcelo, 2020; Jordan et al., 2020; Pfattheicher et al., 2020). Hence, our results suggest that the first phenomenon—social distancing as a justice-related prosocial behavior—was more salient. This explains why recent research has focused on the linkage between social distancing and empathy for vulnerable others (e.g., Pfattheicher et al., 2020) and why some research found that self-protection plays a subordinate role in predicting social distancing (e.g., Twardawski et al., 2021). Thus, regarding social distancing in the COVID-19 pandemic, the other-protective component seems to be of greater relevance. Importantly, evidence only suggests that BJW is related to prosocial outcomes, but not per se to a prosocial motive. Following just world logic, prosocial behavior generally does not necessarily stem from purely altruistic motives but rather from an egoistic expectation of earning subsequent rewards for oneself, for instance in terms of reciprocity by being treated fairly by others in the long term (Dalbert, 2002; Sutton et al., 2017; Zuckerman, 1975).

To shed further light on the role of just world beliefs in predicting social distancing, it is essential to discuss the results of the mediation and exploratory moderation analyses. Although there were plausible arguments to exploratorily assume mediating influences of empathy and risk perception on the relationship between personal BJW and social distancing (see the derivation of Hypothesis 4), no such mediation effects could be found. Even though past research already found BJW to be linked to risk perception (e.g., Furnham, 2003; Lambert et al., 1999; Riley & Baah-Odoom, 2012), this linkage was not evident in our data. Also empathy was not correlated with BJW; thus, undermining mediational effects of perceived infection probability and empathy on the association between personal BJW and social distancing. However, there were some significant correlations between social distancing and risk perception/ empathy, respectively. These correlational findings irrespective of BJW suggest that risk perception and empathy both play a role in people’s social distancing in the way that the more people perceived COVID-19 as a general health threat and the higher people perceived their personal severity of infection, the more they engaged in social distancing, respectively. Again, this indicates that—apart from the other-protective component—there might be an (additional) motive of personal protection behind social distancing. This is in line with some researchers highlighting a positive link between prevention intentions and perception of personal and of public threat (Jordan et al., 2020), but not with others (van Rooij et al., 2020). Note that infection probability did not correlate with social distancing which could be explained by opposing processes: On one hand, people with low probability perceptions could engage less in social distancing, while on the other hand low probability perceptions could be caused by engaging in more social distancing. Overall, the results regarding the role of risk perception in social distancing need further investigation, for instance by using different assessment methods. As our work focuses on the role of BJW in predicting social distancing, risk perception only plays a subordinate role in this specific question, as all measures of risk perception are uncorrelated to people’s BJW. However, one further, explorative finding is worth mentioning: Through our exploratory moderation analysis, we know that when the perceived infection probability is low or moderate, personal BJW can increase social distancing. However, in our study, there was no moderating effect of people’s perceived infection severity nor of the combined measure of risk perception. Besides, as already mentioned, we only conducted the moderation analyses as an exploratory refinement of our hypotheses, and the finding may be an accidental product regarding the number of statistical analyses (multiple testing problem; Sainani, 2009). Hence, also regarding the results from the mediation analyses, these findings should be interpreted with caution and taken up by future research investigating the role of risk perception on social distancing closely to derive more concrete implications. In sum, even though our study suggests that people’s risk perception operates independently of people’s BJW, it may be an important factor in predicting social distancing behavior in the COVID-19 pandemic.

Furthermore, our study suggests that although personal experiences with COVID-19 did not predict social distancing in our regression analyses, they might have an influence on risk perception as we found people with personal COVID-19 experiences (i.e., participants who belonged to a risk group, have been infected themselves or know someone who has been infected or died) to report a higher perception of infection probability as well as a higher perception of infection severity compared to participants without personal experiences (see Further Results Section 3.6 in the Supplemental Material). Future research should therefore explore which variables might influence people’s risk perception in the COVID-19 pandemic.

As indicated above, also empathy showed some results irrespective of people’s BJW. The regression models suggested that while personal BJW and empathy could each explain general social distancing, empathy was descriptively the stronger predictor. Hence, our results replicated the findings by Pfattheicher et al. (2020) providing further support for a positive linkage between empathy for vulnerable others and social distancing behavior.

In addition, our study offers more insight into the well-established distinction between personal BJW and general BJW. More broadly, our findings support Furnham’s (2003) argumentation that although BJW has traditionally been linked to more negative variables like victim blaming, recent research has increasingly emphasized the beneficial functions especially of personal BJW; such as more compliance with social distancing in this case. Also a recent study by Devereux et al. (2021) showed BJW to be linked to more adherence to general containment behaviors in the pandemic, while Gratz et al. (2021) found no linkage between BJW and social distancing. However, these two studies did not distinguish between the two BJW facets. Indicating an impact of personal BJW—not general BJW—in line with our hypotheses, our study therefore highlights the importance of such a BJW distinction and appeals to future studies to do the same.

### Limitations

First, we want to highlight that our study is correlational and solely indicates that personal BJW is associated with higher self-reports of general social distancing. Second, the effects were small. Our study was mainly about testing the theory and less about the actual strength or possible background of the relationship. Hence, it is not possible to draw causal inferences and as our exploratory mediation and moderation analyses suggest, future research should be open to the direction of causal relationships and to different potential mediating and moderating factors which need further investigation (Fiedler et al., 2018).

Third, it is striking that the reported relationship could only be found when social distancing was measured generally and that the effects vanished regarding concrete social distancing. Thereby, the null-findings concerning concrete social distancing must be interpreted with caution regarding the only moderate internal consistency of this scale. Furthermore, the moderate intercorrelation of the two measures indicates that they measured a similar but not the exact same construct. The more general scale assessed participants’ behavior but also their attitude toward other people’s behavior, while the more concrete scale assessed participants’ own intended behavior. Attitudes and behaviors as well as perceptions of oneself and of others are often different from each other, which might be reflected in the differences in people’s responses. Note, only the item concerning participants’ attitude toward general social distancing was significantly linked to personal BJW and not the behavioral one; thus, highlighting the beforementioned conclusion. More importantly, because we aimed at assessing people’s general way of acting during the COVID-19 pandemic in terms of social distancing, the scale concerning concrete social distancing was too specific and too context-dependent for measuring these traits; for instance, by presuming the weather to be good the next few days when the study was conducted over several weeks. Hence, the scale rather measured people’s momentary behavior, or more specifically their momentary behavioral intentions, which however have been shown to vary widely and to not correspond to people’s average behavior (Fleeson, 2004; Schindler & Pfattheicher, 2021). Retrospectively, it would have been a better choice to employ a different scale targeting people’s typical behavior─not susceptible to situational factors─which is the reason why we relocated the according results to the Supplemental Material.

Fourth, the relationship between personal BJW and general social distancing was not significant when excluding participants with personal COVID-19 experience, but this might be due to the reduced sample size and thus reduced power. Interestingly, the exploratory subsample solely consisting of people with personal COVID-19 experiences showed the same correlations as the original sample, thus, supporting the aforementioned findings.

## Conclusion

To our knowledge, the present study was the first one integrating people’s BJW to the discussion about interindividual differences in social distancing during the COVID-19 pandemic. We found initial support that the higher people’s personal BJW, the more they generally engaged in social distancing. This relationship remained robust when controlling for general BJW, empathy, age, gender, perceived infection probability, and personal experiences with COVID-19. Therefore, personal BJW can be regarded as a valuable addition to explaining people’s compliance with social distancing. Independent of BJW, we found further support for the role of empathy in social distancing, and our study suggests that also people’s risk perception might influence social distancing. Overall, the results could help to effectively appeal to people to engage in social distancing by explicitly targeting their personal BJW (not their general BJW). Accordingly, messages should highlight people’s belief that the world is just for oneself. Furthermore, targeting people’s empathy for vulnerable others could help to increase their willingness to socially distance themselves from others. These findings extend past research findings on effective communication during the pandemic (e.g., Blagov, 2021; Heffner et al., 2020; Jordan et al., 2020). Summarizing, our research offers additional ideas for practical implementations, which need to be expanded and tested in future studies focusing on further concrete action mechanisms.

## Footnotes

1 The study was pre-registered with AsPredicted (see #39656; https://aspredicted.org/ek5mp.pdf).
